# Development of a mathematical model to predict the health impact and duration of SARS-CoV-2 outbreaks on board cargo vessels

**DOI:** 10.1007/s13437-022-00291-1

**Published:** 2022-10-19

**Authors:** Kok Yew Ng, Tudor A. Codreanu, Meei Mei Gui, Pardis Biglarbeigi, Dewar Finlay, James McLaughlin

**Affiliations:** 1 Engineering Research Institute, Ulster University, York Street, Belfast, BT15 1ED UK; 2 Electrical and Computer Systems Engineering, School of Engineering, Monash University Malaysia, Jalan Lagoon Selatan, Bandar Sunway, 47500 Selangor, Malaysia; 3 State Health Incident Coordination Centre, Department of Health Western Australia, Perth, Western Australia Australia; 4 Disaster Preparedness and Management Unit, Divisional Commander, Complex Medical Deployments, Department of Health Western Australia, Perth, Western Australia Australia; 5 School of Chemistry and Chemical Engineering, Queen’s University Belfast, David Keir Building, Stranmillis Road, Belfast, BT9 5AG UK; 6 Flowminder Foundation, Regus, Cumberland House, Grosvenor Square, Southampton, SO15 2BG UK

**Keywords:** COVID-19, Coronavirus, Mathematical modelling, Cargo vessels, Western Australia

## Abstract

The Coronavirus Disease (COVID-19) pandemic has brought significant impact onto the maritime activities worldwide, including disruption to global trade and supply chains. The ability to predict the evolution and duration of a COVID-19 outbreak on cargo vessels would inform a more nuanced response to the event and provide a more precise return-to-trade date. This paper presents the SEIQ(H)R (Susceptibility–Exposed–Infected–Quarantine–(Hospitalisation)–Removed/Recovered) model, which is the first deterministic mathematical model developed and fit-tested to describe the transmission dynamics of COVID-19 on board cargo vessels of up to 60 crew members. Due to specific living and working circumstances on board cargo vessels, instead of utilising the reproduction number, we consider the highest fraction of crew members who share the same nationality to quantify the transmissibility of the disease. The performance of the model is verified using case studies based on data collected during COVID-19 outbreaks on three cargo vessels in Western Australia during 2020. The simulations show that the model can forecast the time taken for the transmission dynamics on each vessel to reach their equilibriums, providing informed predictions on the evolution of the outbreak, including hospitalisation rates and duration. The model demonstrates that (a) all crew members are susceptible to infection; (b) their roles on board are a determining factor in the evolution of the outbreak; and (c) an unmitigated outbreak could affect the entire crew and continue on for many weeks. The ability to model the evolution of an outbreak, in both duration and severity, is essential to predict outcomes and to plan for the best response strategy. At the same time, it offers a higher degree of certainty regarding the return to trade, which is of significant importance to multiple stakeholders.

## Introduction

Since the declaration of the Coronavirus Disease 2019 (COVID-19) pandemic by the World Health Organization (WHO) (2020b), merchant vessels worldwide have been affected by Severe Acute Respiratory Syndrome Coronavirus 2 (SARS-CoV-2) outbreaks and ports remain a high-risk entry point for the infection (Codreanu and Armstrong 2021; Codreanu et al. 2021a, b). The spread of infectious diseases amongst crew on board cargo vessels is facilitated by several factors including a high population density in frequent direct contact areas, sharing of general amenities on board (Codreanu et al. 2021b; World Health Organization 2020a), and interactions with shore-based maritime workers. Risk mitigation through international sanitation legislation requirements (World Health Organization 2011a, b; 2016; EU EUSHIPSAN Act Joint Action 2016; World Health Organization 2005) and the measures mandated under the International Health Regulations (Codreanu et al. 2021b; Rocklöv et al. 2020; Tabata et al. 2020; Nakazawa et al. 2020) have decreased the transmissions of SARS-CoV-2 (Codreanu et al. 2021a, b; Rocklöv et al. 2020), but were unable to fully control outbreaks on board (Codreanu and Armstrong 2021; Codreanu et al. 2021a; Walker et al. 2021).

Unlike cruise ships that accommodate very large numbers of passengers and crew (Codreanu et al. 2021a), cargo vessels operate with a comparatively much fewer crew (Codreanu et al. 2021b), and with very different work and social interaction patterns. Granting of pratique is based on providing objective and subjective data of the situation on board prior to arrival, of which assessment could be misleading (Codreanu and Armstrong 2021). The declaration of an outbreak on board a cargo vessel has implications not only for the crew, but also for the vessel owner, shipping agent, operator, flag state, trade partners, and incident response agencies. Notwithstanding the threat to the crew’s health (Codreanu et al. 2021a; Schlaich et al. 2009), outbreak management not only has significant logistic and economic impact on the response agencies, but also on maritime transport and trade (Codreanu et al. 2021b; Jerome et al. 2017). In addition, the containment measures (quarantine, isolation, and sanitation), travel restrictions, and border closures (Australian Government 2015) continue to make it increasingly difficult for ship operators worldwide to be granted pratique (Dujarric 2015; Australian Government Department of Health 2020), conduct trade, and change crew (Australian Government Department of Agriculture Water and Environment 2020).

The Susceptibility–Infected–Removed (SIR) or Susceptibility–Exposed–Infected–Removed (SEIR) mathematical models have been used to describe the transmission dynamics of infectious diseases outbreaks (Kermack and McKendrick 1927) in which the epidemic is represented in a series of separate compartments or subpopulations. The SEIR model was used to predict the effectiveness of public health measures to control the COVID-19 outbreak on a passenger cruise ship (Rocklöv et al. 2020), and a modified SEIR model that includes hospitalisation, quarantine, and isolation was used to model other infectious disease epidemics (Safi and Gumel 2010; 2011).

This paper presents the first deterministic mathematical model to predict the transmission dynamics of COVID-19 on board cargo vessels of up to 60 crew members. The generalised SEIQ(H)R model was developed and modified based on previous reported works (Safi and Gumel 2010; 2011; Ng and Gui 2020; Do et al. 2021) and represents the epidemic in separate compartments in cascade: susceptibility (S), exposed (E), infected (I), quarantine (Q), hospitalisation (H), and removed/recovered (R). This research is crucial towards the fight against the COVID-19 pandemic as we address the importance of modelling the transmission dynamics of SARS-CoV-2 amongst crew members on board cargo vessels, which has rarely been considered in published works thus far as most of them reported on passenger cruise ships (Rocklöv et al. 2020; Mizumoto and Chowell 2020; Mizumoto et al. 2020).

The continuous advancement of knowledge on the clinical outcomes of COVID-19 and the role of associated factors such as age, co-morbidities, and ethnicity (Clift et al. 2020; Lighter et al. 2020) should underpin a more nuanced approach to managing outbreaks on cargo vessels in order to minimise the impact on crew health, maritime traffic, and trade. As a result, mathematical models can be used to provide an accurate and dynamic prediction of a COVID-19 outbreak progression in this setting.

Knowledge of the modelled evolution of the COVID-19 outbreak, both in duration and severity, is essential to predict outcomes and to plan for the most efficient and effective response strategies. Based on the Western Australian experience in managing COVID-19 outbreaks on board cargo vessels during 2020, here we describe the development of a mathematical model that could be used to inform the risk assessment of the population-specific epidemiological parameters for SARS-CoV-2 spread on similar vessels of up to 60 crew. This research is also important as the predictions from the model would help stakeholders in the analysis and understanding of the transmission dynamics of an infection disease on board a vessel during the earlier stages of a pandemic, especially when a vaccine has yet to be developed or that the availability of vaccine is scarce. Hence, maritime operations during this period are most vulnerable to the impact of the pandemic with lack of quality testing facilities, as well as the imposition of quarantines and closure of international borders and ports. Using this model, the responders to the outbreak will have a common understanding of the event evolution, including hospitalisation rates, as a function of the measures implemented.

This paper is organised as follows: Section 2 introduces the SEIQ(H)R mathematical model; Section 3 describes the data sources of the three cargo vessels used to verify and validate the proposed mathematical model; Section 4 verifies the proposed model and also provides some extensive results and discussions about the simulations; Section 5 presents the limitations of this research; and Section 6 concludes the paper. The outbreak data of the cargo vessels can be found in the Appendix.

## Methodology

The SEIQ(H)R model was developed utilising data recorded during quarantine measures and hospitalisations on board the vessels, and the following assumptions: (i) absence of vaccinations against SARS-CoV-2, (ii) absence of confirmed history of previous COVID-19 infections, and (iii) absence of non-COVID-19-related deaths occurring on the vessels. Hence, the model can be expressed using

1 $$\begin{array}{@{}rcl@{}} \dot{p}_{S} & =& -\beta_{e} p_{S}p_{I}, \end{array}$$

2 $$\begin{array}{@{}rcl@{}} \dot{p}_{E} & =& \beta_{e} p_{S}p_{I} - \alpha p_{E}, \end{array}$$

3 $$\begin{array}{@{}rcl@{}} \dot{p}_{I} & =& \alpha p_{E} - \lambda p_{I}, \end{array}$$

4 $$\begin{array}{@{}rcl@{}} \dot{p}_{Q} & =& \lambda p_{I} + \kappa p_{H} - \omega p_{Q} - \gamma p_{Q}, \end{array}$$

5 $$\begin{array}{@{}rcl@{}} \dot{p}_{H} & =& \omega p_{Q} - \kappa p_{H}, \end{array}$$

6 $$\begin{array}{@{}rcl@{}} \dot{p}_{R} & =& \gamma p_{Q}, \end{array}$$

where *p*_*S*_, *p*_*E*_, *p*_*I*_, *p*_*Q*_, *p*_*H*_, and *p*_*R*_ represent the susceptible (S), exposed (E), infectious (I), quarantined (Q), hospitalised (H), and removed/recovered (R) subpopulations, respectively.^1^ The parameter *β*_*e*_ denotes the rate of transmission per S-I contact, *α* the rate of an exposed person becoming infectious, *λ* the rate of which an infectious person is quarantined, *ω* the rate of which a quarantined person is hospitalised, *γ* the rate of which a quarantined person recovers, and *κ* is the rate of which a hospitalised person is discharged and completes their quarantine.

Thus, the incubation time can be written as *τ*_*inc*_ = 1/*α*, the time from onset to being quarantined is *τ*_*infQ*_ = 1/*λ*, the time spent in quarantine before recovery is *τ*_*quarR*_ = 1/*γ*, the time spent in quarantine before being hospitalised is *τ*_*quarH*_ = 1/*ω*, and the hospitalisation time is *τ*_*hosp*_ = 1/*κ*. Overall, the total number of crew members on the vessel, *p*_*N*_, can be computed such that

7 $$p_{N} = p_{S}+p_{E}+p_{I}+p_{Q}+p_{H}+p_{R},$$

which indicates that at any given time, the number of crew on board the vessel would remain the same, irrespective of their health conditions.

**Fig. 1:**
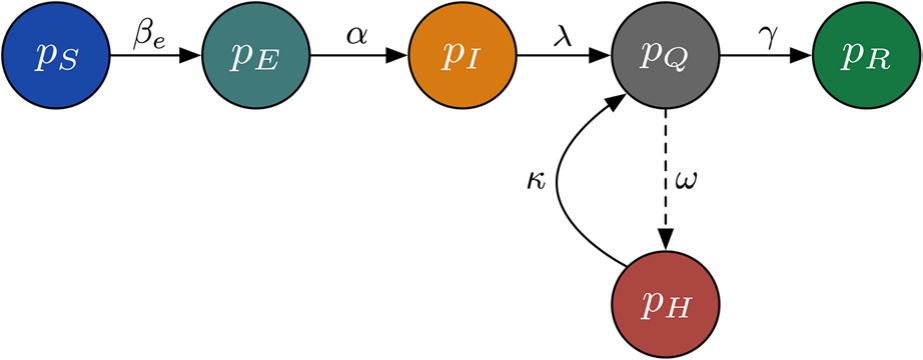
The block diagram of the SEIQ(H)R model in Eqs. (1)–(6). The arrows indicate the directions of which the crew members would move from one compartment (or subpopulation) to another as they progress through the transmission dynamics of SARS-CoV-2. The dashed arrow indicates an optional path of which a quarantined crew would be hospitalised, as not all who are quarantined have to be hospitalised

Given the crew size, the transmission dynamics and spread of the infectious disease cannot be accurately quantified using the reproduction number that is commonly applied to studies involving large populations, i.e. a country or a geographical region. Thus, instead of using the parameter *β*_*e*_ (or its equivalent mathematical representation in other existing models) to compute the reproduction number (Safi and Gumel 2010; 2011; Ng and Gui 2020; Do et al. 2021), its purpose here is to inform the transmission dynamics of SARS-CoV-2 by considering the majority of crew members who share the same nationality. This information is estimated critical as crew who originate from the same country often share similar language and culture, thus have increased social interactions on board, which in turn would reasonably be expected to increase the rate of transmission. Therefore, in this paper, *β*_*e*_ is expressed using

8 $$\beta_{e} = km_{p},$$

where *m*_*p*_ represents the highest fraction of crew who originate from the same country and *k* is a scalar coefficient and design freedom. All variables and parameters of the model shown in Eqs. (1)–(8) are tabulated in Table 1. Figure 1 shows the block diagram of the SEIQ(H)R model in Eqs. (1)–(6), where the arrows indicate the directions of which the crew members would move from one compartment (or subpopulation) to another as they progress through the transmission dynamics of SARS-CoV-2. The dashed arrow indicates an optional path of which a quarantined crew would be hospitalised, as not all who are quarantined have to be hospitalised.

**Table 1:** List of variables and parameters of the SEIQ(H)R model formulated in Eqs. (1)–(8)

Variable/Parameter	Description
*p*_*N*_	Total number of crew members on board vessel
*p*_*S*_	Crew members who are susceptible to SARS-CoV-2 (S)
*p*_*E*_	Crew members who are exposed to SARS-CoV-2 (E)
*p*_*I*_	Crew members who are infectious (I)
*p*_*Q*_	Crew members who are in quarantine due to SARS-CoV-2 (Q)
*p*_*H*_	Crew members who are hospitalised due to SARS-CoV-2 (H)
*p*_*R*_	Crew members who have recovered from SARS-CoV-2 (R)
$\dot {p}_{S}$	Rate of change of susceptible crew members
$\dot {p}_{E}$	Rate of change of exposed crew members
$\dot {p}_{I}$	Rate of change of infectious crew members
$\dot {p}_{Q}$	Rate of change of quarantined crew members
$\dot {p}_{H}$	Rate of change of hospitalised crew members
$\dot {p}_{R}$	Rate of change of recovered crew members
*β*_*e*_	Rate of a susceptible crew member becoming exposed
*m*_*p*_	Highest fraction of crew members who share the same nationality
*k*	Scalar coefficient and design freedom
*α*	Rate of an exposed crew member becoming infectious
*λ*	Rate of an infectious crew member being quarantined
*γ*	Rate of a quarantined crew member to recover from SARS-CoV-2
*ω*	Rate of a quarantined crew member is hospitalised
*κ*	Rate of a hospitalised crew member to be discharged
*τ*_*inc*_	Incubation time, 1/*α*
*τ*_*infQ*_	Time from onset to being quarantined, 1/*λ*
*τ*_*quarR*_	Time spent in quarantine before recovery, 1/*γ*
*τ*_*quarH*_	Time spent in quarantine before being hospitalised, 1/*ω*
*τ*_*hosp*_	Hospitalisation time, 1/*κ*

## Data sources

The data used are based on the records of COVID-19 outbreaks declared on the cargo vessels MV Al Kuwait, MV Al Messilah, and MV Patricia Oldendorff during 2020 in Western Australia.

The data collection, analysis, storage, and reporting were conducted in line with the WHO Ethical Standards for Research During Public Health Emergencies (COVID-19), the WHO Guidance for Managing Ethical Issues in Infectious Diseases Outbreaks, and the WHO Guidelines on Ethical Issues in Public Health Surveillance. Ethics approval was not required for this investigation as it was conducted as part of the public health response to the outbreaks of COVID-19, which is a notifiable infectious disease under the Western Australia Public Health Act 2016. The release of data not already in the public domain has been granted by the Western Australia Department of Health Public Health Emergency Operations Centre Data Custodian. The Western Australian Department of Health Human Research Ethics Committee (HREC) has determined that the routine public health investigative work relating to a series of outbreaks of infectious disease that involved aggregate data, in accordance with Section 5.1.8 of the National Statement, involves only negligible risk (i.e. where there is no foreseeable risk or harm or discomfort, and any foreseeable risk is no more than inconvenience) and therefore does not need to be reviewed by the HREC.

## Results and discussion

In aggregate, 63 out of 122 crew were diagnosed with COVID-19 (51.64%), and three (4.76%) of the cases required hospitalisation, but not mechanical ventilatory support. Table 2 summarises the data from the three vessels (additional data are provided in the Appendix).

**Table 2:** Summarised data of the three vessels

Description	Value
**Vessel 1: MV Al Kuwait**	
Total crew members	48
*Crew members by nationalities*	
Philippines	32 (66.67%)
Croatia	10 (20.83%)
India	3 (6.25%)
Australia	2 (4.17%)
Tanzania	1 (2.08%)
Total confirmed cases	21
Total hospitalised	2
Mean quarantine days	16.4
Mean hospitalisation days	2
**Vessel 2: MV Al Messilah**	
Total crew members	53
*Crew members by nationalities*	
Bangladesh	37 (69.81%)
India	7 (13.21%)
Sri Lanka	4 (7.54%)
Pakistan	3 (5.66%)
Australia	1 (1.89%)
Syria	1 (1.89%)
Total confirmed cases	25
Total hospitalised	1
Mean quarantine days	10.5
Mean hospitalisation days	2
**Vessel 3: MV Patricia Oldendorff**	
Total crew members	21
* Crew members by nationalities*	
Philippines	20 (95.24%)
Poland	1 (4.76%)
Total confirmed cases	17
Total hospitalised	0
Mean quarantine days	9.8
Mean hospitalisation days	–

Simulations to verify the model were carried out using MATLAB/Simulink R2021a.^2^ Given the data in Table 2 as well as the full records provided in the Appendix, and to ensure that the model can be generalised, the parameters of the model in Eqs. (1)–(6) for the three cargo vessels are set as shown in Table 3. The parameter *m*_*p*_ is determined by the highest fraction of nationality in each vessel. The scalar coefficient *k* is a design freedom and is set to 1.15 for all three vessels to indicate that it does not need to be tuned and can be applied generally to cargo vessels of similar settings. The rate of an exposed crew becoming infectious *α* is the reciprocal of the incubation period and is set to 1/5 in accordance to Flaxman et al. (2020). The remaining parameters in Table 3, i.e. *λ*, *γ*, *ω*, and *κ*, are set using the mean values obtained from the data.

**Table 3:** Parameters settings of the SEIQ(H)R model for the three cargo vessels used in the case studies

Parameter	Description	MV Al Kuwait	MV Al Messilah	MV Patricia Oldendorff
*m*_*p*_	Highest fraction of crew who share the same nationality	0.67	0.70	0.95
*k*	Scalar coefficient	1.15	1.15	1.15
*α*	Rate of an exposed crew becoming infectious (5 days)	1/5	1/5	1/5
*λ*	Rate of an infectious crew being quarantined (5 days)	1/5	1/5	1/5
*γ*	Rate of a quarantined crew recovers (95%, 15 days)	0.95 × 1/15	0.95 × 1/15	0.95 × 1/15
*ω*	Rate of a quarantined crew is hospitalised (5%, 2 days)	0.05 × 1/2	0.05 × 1/2	0.05 × 1/2
*κ*	Rate of a hospitalised crew is discharged (2 days)	1/2	1/2	1/2

**Fig. 2:**
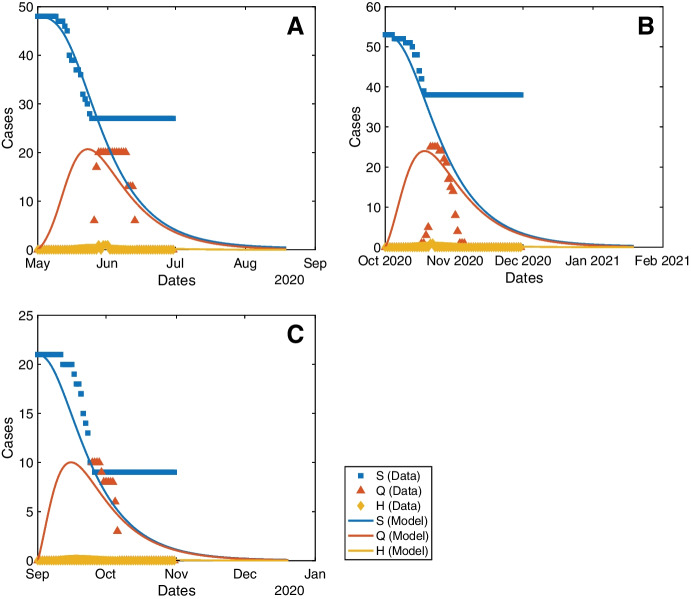
Data and model plots for the S, Q, and H compartments of vessels (A) MV Al Kuwait, (B) MV Al Messilah, and (C) MV Patricia Oldendorff

The convergence between the results of the proposed model and the observed data for each vessel are presented in Fig. 2, which shows that the simulations can forecast the time for the transmission dynamics on each vessel to reach their equilibriums. Thus, these results are able to provide informed predictions on the evolution of the outbreak on board that would be useful to determine if and when the vessel can be granted pratique to conduct trade and crew exchange.

The model also suggests that all crew members should be considered susceptible to infection and that, if left unmitigated, the transmission of the infection on board can

- affect the entire crew, potentially crippling the safety and security of the vessel by breaching the mandated Minimum Safe Manning (Codreanu et al. 2021b), and
- continue on for many weeks, contrary to estimations declared by some operators (Codreanu and Armstrong 2021)

The very low number and short duration of hospital admissions could reflect the relative absence of pre-existing or underlying health risk factors in the individuals affected, which supports a nuanced approach to the management of the outbreak. However, the risks of severe disease and death are not null, and a clinical deterioration at sea can have catastrophic consequences to the individuals, the larger crew, and the vessel. This is further complicated by the default absence of medical facilities, monitoring equipment, and qualified medical personnel on board cargo vessels.

**Fig. 3:**
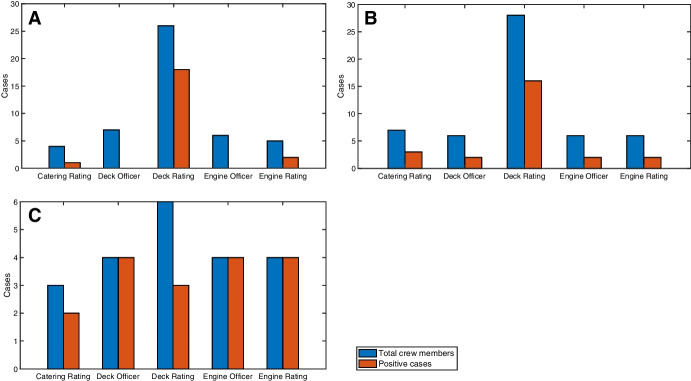
Cases by crew occupation (officers and ratings) for vessels (A) MV Al Kuwait, (B) MV Al Messilah, and (C) MV Patricia Oldendorff. The Catering Ratings are a common intersection point for all vessels

It was also found that crew members’ roles on board is a determining factor in the evolution of the outbreak (Fig. 3). This could be due to the characteristics of the accommodation structure on cargo vessels that include common predicted intersection points (e.g. mess rooms and catering staff, as well as shared facilities such as showers and toilets) irrespective of the technical segregation between officers and ratings, in both the deck and engine groups. This is evidenced in Fig. 3A where only three groups of crew occupations have tested positive. Given that the Deck Rating crew have limited physical interactions with the Engine Rating crew and also that they work in different sections of the vessel, it can be assumed that the Catering Ratings are a common intersection point in the transmission of the infection.

**Fig. 4:**
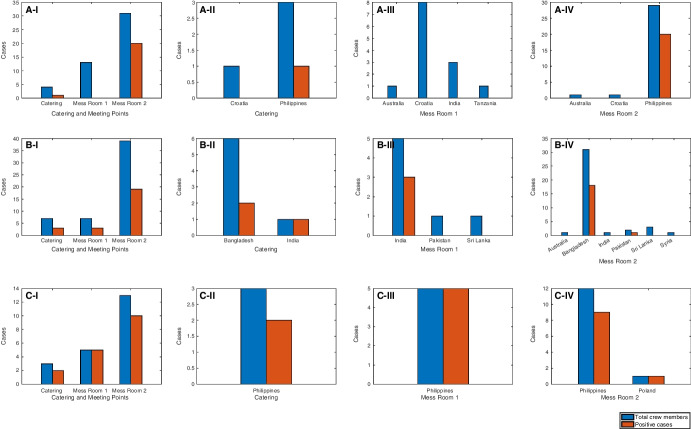
Total crew members (blue) and positive cases (red) in Catering, Mess Room 1, and Mess Room 2 intersection points for vessels (A) MV Al Kuwait, (B) MV Al Messilah, and (C) MV Patricia Oldendorff, respectively. Subplots (I) show the collective data for each vessel whilst subplots (II)–(IV) show the distribution of each intersection point by nationality

The Maritime Labour Convention, 2006 (International Labour Organization 2006) requires that separate mess room facilities are provided to crew members of different ranking: (i) master and officers, and (ii) petty officers and other crew. Thus, officers and higher ranked crew members would use Mess Room 1, and the rest of the crew, Mess Room 2. However, all hot meals are prepared and delivered by the same catering staff. Figure 4 demonstrates that, on each vessel, there were higher number of positive cases recorded in crew from the same nationality (or closely related cultures) with the catering staff. These observations agree with the hypothesis that the more similar the culture and language, irrespective of nationality, the more social interactions occur on board, which, in turn, increases the transmission of the virus.

## Limitations

The proposed model fits the data collected during the management of the three COVID-19 outbreaks described and is limited to a typical cargo vessel crew size of no more than 60 crew members. More data would be required to ascertain that the observed model and data convergence can be generalised to larger crew sizes. The model has not been calibrated for complex individualist versus collectivist cultural aspects of the crew, nor for social interaction patterns on board as these are largely superseded by clear hierarchical command and control relationships as well as work-related intersection points. Therefore, further division of the crew in subclasses of susceptible individuals has not been deemed necessary as it is not expected to alter the modelling. In addition, population of passengers are not considered in the mathematical model as the operations of cargo vessels differ significantly from passenger cruise vessels.

Also, vaccines against SARS-CoV-2 were not available at the time of the study. Whilst noting that the main aim of vaccines is to reduce the risk of severe disease and death, and not necessarily the susceptibility to infection, vaccination status is crucial in the modelling design as the predictions would help stakeholders in the analysis and understanding of the transmission dynamics of an infection disease on board a cargo vessel. Early stages of any outbreaks with little known pathogens and adequate mitigation strategies are also the periods of which maritime operations are most affected: absence of readily accessible testing platforms and/or facilities, impost of quarantine of varying lengths, and unpredictable duration of closure of international borders and ports. Modelling works may not be as relevant during the later stages of the pandemic when most population would have been protected by the development of vaccines or natural herd immunity. However, the continuous emergence of variants of concern which may result in reinfections or evade the enhanced immunity provided by current vaccines, and the inequal availability of vaccines around the world provides enough reasons for the enduring validity of this mathematical concept.

The model does not consider individual’s past COVID-19 illness as quality information is usually not available at the time of the outbreak declaration. Since gathering and verifying the crew’s previous COVID-19 infection data may prove logistically challenging where reliable information is lacking, serological determination may be required that prolongs the timeline to verification. Further refining of the model could be undertaken by incorporating emerging evidences regarding the protection to SARS-CoV-2 (re)infection in vaccinated individuals.

## Conclusion

COVID-19 outbreaks on cargo vessels continue to present significant economic, trade, and health impacts to countries worldwide (United Nations Conference on Trade and Development (UNCTAD) 2021; Goodman 2022; Murray et al. 2022). The ability to model the evolution of an outbreak on board typical crew-sized cargo vessels, both in duration and severity, is essential to predict outcomes and to plan for the best response strategies. This paper has presented, to the best of the authors’ knowledge, the first deterministic mathematical model developed to simulate the transmission dynamics of SARS-CoV-2 on board cargo vessels with up to 60 crew members. Using limited health-related data gathered about the crew on board three cargo vessels in Western Australia in 2020, the SEIQ(H)R model proposed in this paper was able to provide a good fit for the outbreaks on the vessels. Most crucially, the model was able to predict the time taken for the transmission dynamics of SARS-CoV-2 on each vessel to reach their equilibriums, hence capable of providing informed predictions on the evolution of the outbreak, including hospitalisation rates and duration.

Future works include further modifications and refinements to this model that would allow for simulations and forecasts of new SARS-CoV-2 variants or even other existing or new infectious diseases on board cargo vessels in the future. With that, the proposed model can be used by policymakers and governing bodies in planning for the necessary control actions to reduce or even mitigate the spread of potential future outbreaks, e.g. to decide on an optimum duration of quarantine/isolation, to predict the development of any new outbreaks, to estimate hospitalisation numbers and duration, etc. Further research is required to determine its generalisability onto other classes and categories of cargo vessels and crew sizes, whilst also focussing on new and emerging SARS-CoV-2 variants, as well as previous COVID-19 disease and/or vaccination status.

## Appendix: . Outbreak data of cargo vessels

The data for all three cargo vessels used in the case studies are presented in Tables 4, 5, and 6.

### A.1 Vessel 1: MV Al Kuwait

Table 4

**Table 4:** Data for Vessel MV Al Kuwait

Crew	Rank (Generalised)	Age Group	Confirmed Case	Onset Date	Quarantine			Hospitalised			
					Start Date	End Date	Days	Yes/No	Admission Date	Discharged Date	Days
1	Engine Officer	35−39y	No								
2	Engine Officer	35−39y	No								
3	Deck Officer	50−54y	No								
4	Deck Rating	40−44y	No								
5	Engine Rating	50−54y	No								
6	Deck Rating	40−44y	No								
7	Deck Rating	45−49y	Yes	14/05/2020	27/05/2020	10/06/2020	14	No			
8	Catering Rating	65−70y	No								
9	Deck Rating	50−54y	Yes	18/05/2020	28/05/2020	13/06/2020	16	No			
10	Deck Rating	45−49y	No								
11	Deck Rating	40−44y	Yes	21/05/2020	28/05/2020	10/06/2020	13	No			
12	Deck Officer	30−34y	No								
13	Deck Rating	40−44y	Yes	13/05/2020	27/05/2020	14/06/2020	18	No			
14	Deck Rating	45−49y	Yes	21/05/2020	27/05/2020	14/06/2020	18	No			
15	Deck Rating	45−49y	No								
16	Catering Rating	45−49y	Yes	18/05/2020	27/05/2020	13/06/2020	17	No			
17	Deck Rating	50−54y	Yes	22/05/2020	26/05/2020	14/06/2020	19	Yes	30/05/2020	02/06/2020	3
18	Deck Rating	40−44y	Yes	15/05/2020	26/05/2020	10/06/2020	15	No			
19	Deck Rating	30−34y	Yes	16/05/2020	26/05/2020	10/06/2020	15	No			
20	Deck Rating	45−49y	No								
21	Deck Rating	60−64y	No								
22	Engine Rating	25−29y	No								
23	Deck Officer	60−64y	No								
24	Deck Rating	40−44y	Yes	24/05/2020	26/05/2020	10/06/2020	15	No			
25	Deck Rating	50−54y	Yes	20/05/2020	27/05/2020	13/06/2020	17	No			
26	Deck Rating	30−34y	Yes	15/05/2020	27/05/2020	14/06/2020	18	No			
27	Deck Rating	40−44y	Yes	15/05/2020	27/05/2020	14/06/2020	18	No			
28	Deck Officer	60−64y	No								
29	Deck Rating	40−44y	Yes	24/05/2020	26/05/2020	13/06/2020	18	No			
30	Deck Rating	30−34y	Yes	10/05/2020 (Remained as EC^1^)				No			
31	Deck Rating	35−39y	Yes	15/05/2020	27/05/2020	13/06/2020	17	No			
32	Deck Rating	50−54y	Yes	15/05/2020	27/05/2020	10/06/2020	14	No			
33	Catering Rating	25−29y	No								
34	Deck Rating	45−49y	Yes	23/05/2020	28/05/2020	10/06/2020	13	No			
35	Deck Officer	60−64y	No								
36	Deck Officer	30−34y	No								
37	Engine Officer	55−59y	No								
38	Engine Officer	50−54y	No								
39	Engine Officer	30−34y	No								
40	Deck Rating	30−34y	Yes	25/05/2020	27/05/2020	14/06/2020	18	No			
41	Deck Rating	35−39y	No								
42	Catering Rating	30−34y	No								
43	Engine Rating	40−44y	No								
44	Engine Rating	30−34y	Yes	21/05/2020	26/05/2020	13/06/2020	18	No			
45	Engine Rating	55−59y	Yes	21/05/2020	27/05/2020	13/06/2020	17	Yes	28/05/2020	29/05/2020	1
46	Engine Officer	60−64y	No								
47	Deck Rating	45−49y	No								
48	Deck Officer	25−29y	No								

^1^Essential crew

### A.2 Vessel 2: MV Al Messilah

Table 5

**Table 5:** Data for Vessel MV Al Messilah

Crew	Rank (Generalised)	Age Group	Confirmed Case	Onset Date	Quarantine			Hospitalised			
					Start Date	End Date	Days	Yes/No	Admission Date	Discharged Date	Days
1	Deck Rating	50−54y	No								
2	Deck Rating	40−44y	No								
3	Engine Officer	55−59y	No								
4	Engine Rating	35−39y	No								
5	Deck Rating	25−29y	Yes	16/10/2020	21/10/2020	02/11/2020	12	No			
6	Engine Rating	25−29y	Yes	18/10/2020	21/10/2020	01/11/2020	11	No			
7	Deck Officer	60−64y	Yes	14/10/2020	21/10/2020	27/10/2020	6	No			
8	Deck Officer	50−54y	No								
9	Deck Rating	50−54y	No								
10	Deck Rating	60−64y	Yes	14/10/2020	21/10/2020	02/11/2020	12	No			
11	Deck Rating	35−39y	Yes	Asymptomatic	21/10/2020	28/10/2020	7	No			
12	Engine Rating	60−64y	Yes	Asymptomatic	21/10/2020	01/11/2020	11	No			
13	Deck Rating	20−24y	No								
14	Catering Rating	25−29y	No								
15	Deck Rating	40−44y	No								
16	Deck Rating	60−64y	No								
17	Deck Rating	45−49y	Yes	Asymptomatic	19/10/2020	29/10/2020	10	No			
18	Catering Rating	25−29y	No								
19	Deck Rating	20−24y	Yes	16/10/2020	21/10/2020	01/11/2020	11	No			
20	Deck Rating	20−24y	Yes	13/10/2020	21/10/2020	27/10/2020	6	No			
21	Catering Rating	25−29y	Yes	16/10/2020	21/10/2020	01/11/2020	11	No			
22	Catering Rating	20−24y	Yes	Asymptomatic	20/10/2020	31/10/2020	11	No			
23	Engine Rating	40−44y	No								
24	Deck Rating	20−24y	No								
25	Catering Rating	25−29y	No								
26	Deck Rating	40−44y	Yes	Asymptomatic	21/10/2020	30/10/2020	9	No			
27	Deck Officer	55−59y	No								
28	Deck Rating	50−54y	No								
29	Engine Officer	25−29y	No								
30	Deck Rating	50−54y	Yes	16/10/2020	21/10/2020	02/11/2020	12	No			
31	Catering Rating	35−39y	Yes	19/10/2020	20/10/2020	03/11/2020	14	Yes	21/10/2020	23/10/2020	2
32	Deck Rating	55−59y	Yes	Asymptomatic	21/10/2020	29/10/2020	8	No			
33	Deck Rating	50−54y	Yes	17/10/2020	21/10/2020	06/11/2020	16	No			
34	Deck Rating	40−44y	No								
35	Deck Rating	40−44y	No								
36	Deck Rating	50−54y	Yes	05/10/2020	21/10/2020	02/11/2020	12	No			
37	Engine Rating	25−29y	No								
38	Deck Officer	40−44y	Yes	18/10/2020	21/10/2020	03/11/2020	13	No			
39	Deck Officer	60−64y	No								
40	Deck Rating	15−19y	No								
41	Deck Officer	45−49y	No								
42	Deck Rating	40−44y	Yes	Asymptomatic	21/10/2020	03/11/2020	13	No			
43	Catering Rating	30−34y	No								
44	Deck Rating	60−64y	Yes	Asymptomatic	21/10/2020	01/11/2020	11	No			
45	Engine Officer	40−44y	No								
46	Deck Rating	40−44y	Yes	17/10/2020	21/10/2020	30/10/2020	9	No			
47	Deck Rating	35−39y	No								
48	Engine Officer	30−34y	No								
49	Deck Rating	15−19y	Yes	10/10/2020	17/10/2020	25/10/2020	8	No			
50	Engine Officer	25−29y	Yes	18/10/2020	19/10/2020	29/10/2020	10	No			
51	Engine Officer	45−49y	Yes	Asymptomatic	21/10/2020	29/10/2020	8	No			
52	Engine Rating	60−64y	No								
53	Deck Rating	50−54y	Yes	Asymptomatic	21/10/2020	01/11/2020	11	No			

### A.3 Vessel 3: MV Patricia Oldendorff

Table 6

**Table 6:** Data for Vessel MV Patricia Oldendorff

Crew	Rank (Generalised)	Age Group	Confirmed Case	Onset Date	Quarantine			Hospitalised			
					Start Date	End Date	Days	Yes/No	Admission Date	Discharged Date	Days
1	Deck Officer	25–29y	Yes	22/09/2020 (Remained as EC^1^)				No			
2	Deck Rating	30–34y	No								
3	Engine Rating	25–29y	Yes	18/09/2020	25/09/2020	29/09/2020	4	No			
4	Engine Officer	30–34y	Yes	12/09/2020 (Remained as EC^1^)				No			
5	Deck Rating	45–49y	No								
6	Engine Rating	20–24y	Yes	24/09/2020	25/09/2020	05/10/2020	10	No			
7	Engine Rating	50–54y	Yes	23/09/2020 (Remained as EC^1^)				No			
8	Engine Officer	20–24y	Yes	24/09/2020	25/09/2020	05/10/2020	10	No			
9	Engine Officer	55–59y	Yes	Asymptomatic (Remained as EC^1^)				No			
10	Deck Officer	20–24y	Yes	21/09/2020	25/09/2020	06/10/2020	11	No			
11	Deck Rating	25–29y	No								
12	Deck Rating	25–29y	Yes	26/09/2020	25/09/2020	07/10/2020	12	No			
13	Deck Officer	55–59y	Yes	Asymptomatic (Remained as EC^1^)				No			
14	Engine Officer	40–44y	Yes	21/09/2020	25/09/2020	06/10/2020	11	No			
15	Catering Rating	35–39y	Yes	17/09/2020	25/09/2020	30/09/2020	5	No			
16	Engine Rating	30–34y	Yes	20/09/2020	25/09/2020	06/10/2020	11	No			
17	Deck Rating	20–24y	Yes	24/09/2020 (Remained as EC^1^)				No			
18	Catering Rating	40–44y	No								
19	Deck Officer	30–34y	Yes	Asymptomatic (Remained as EC^1^)				No			
20	Deck Rating	25–29y	Yes	Asymptomatic	25/09/2020	07/10/2020	12	No			
21	Catering Rating	30–34y	Yes	Asymptomatic	25/09/2020	07/10/2020	12	No			

^1^ Essential crew

## Footnotes

1 Mathematical notation: $\dot {x}$ denotes the first order derivative of the variable *x* with respect to time, i.e. $\dot {x} = \frac {dx}{dt}$.

2 The Simulink model can be made available on request from the authors.
